# Supplementary material for: Discriminant Analysis PCA-LDA Assisted Surface-Enhanced Raman Spectroscopy for Direct Identification of Malaria-Infected Red Blood Cells
Source: Methods Protoc. 2022 Jun 10;5(3):49. doi: 10.3390/mps5030049 (PMC9231316; doi:10.3390/mps5030049)
Supplement: Supplementary file 1 [file mps-05-00049-s001.zip › mps-1696124-SI.pdf]

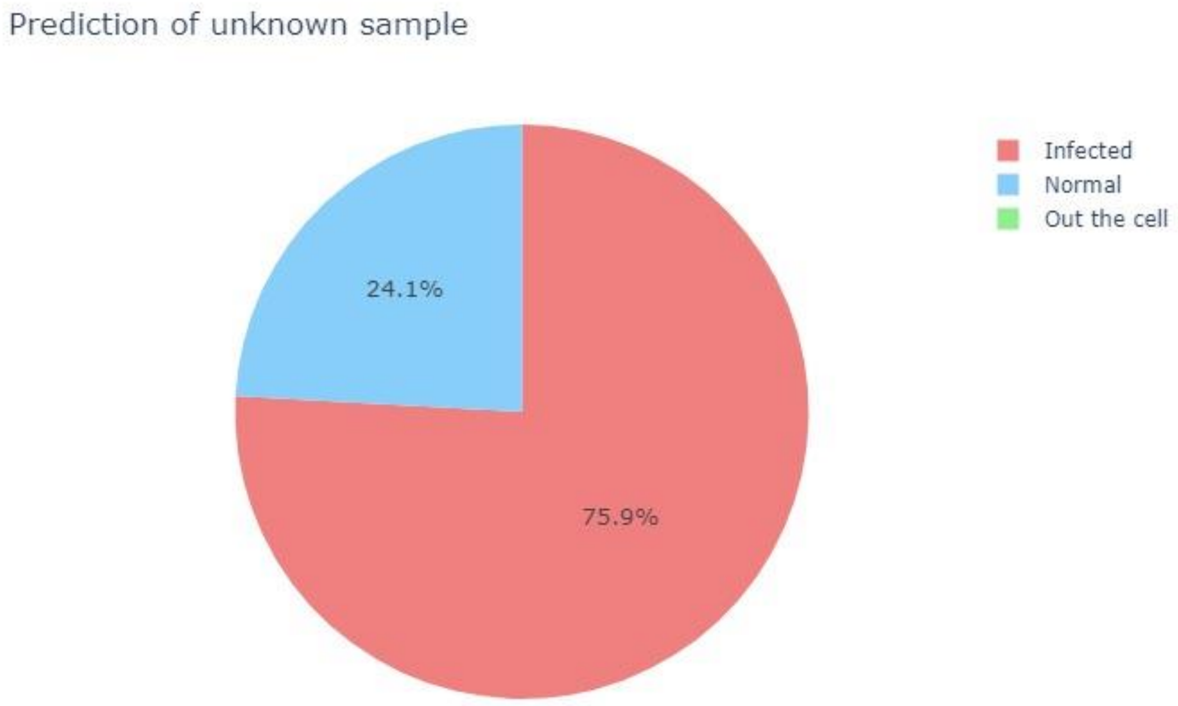

Blind test sample No.1
(The example of the prediction pie chart in the manuscript)

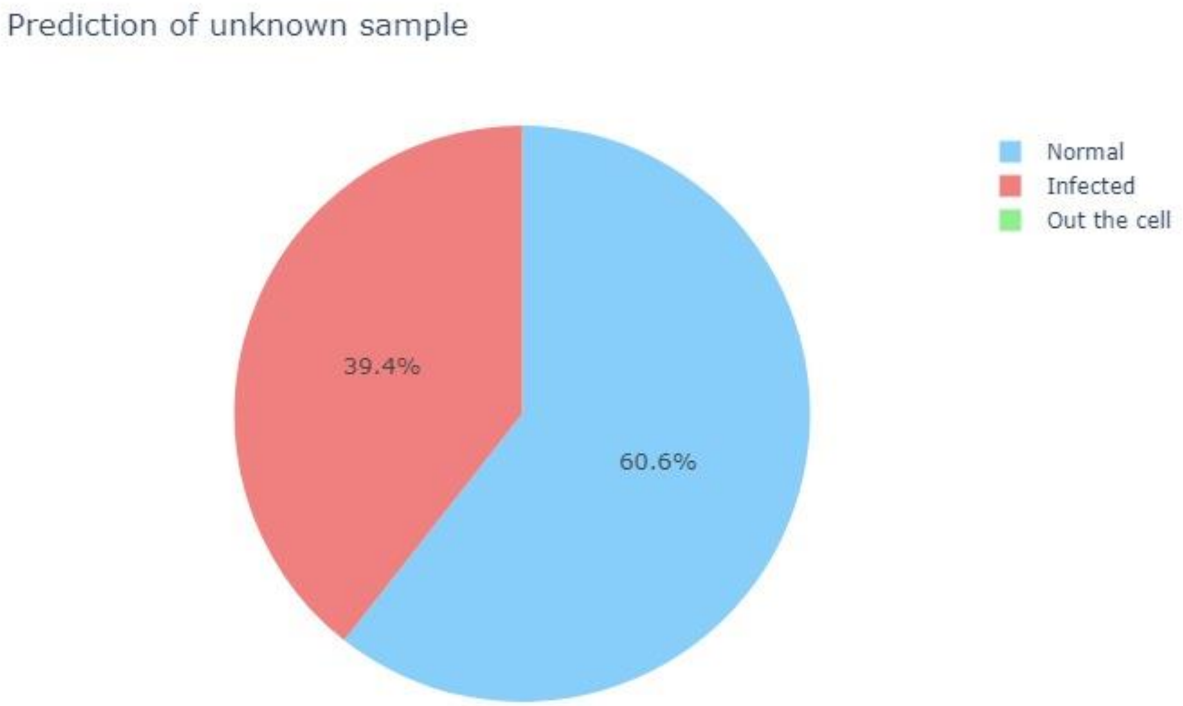

Blind test sample No.2

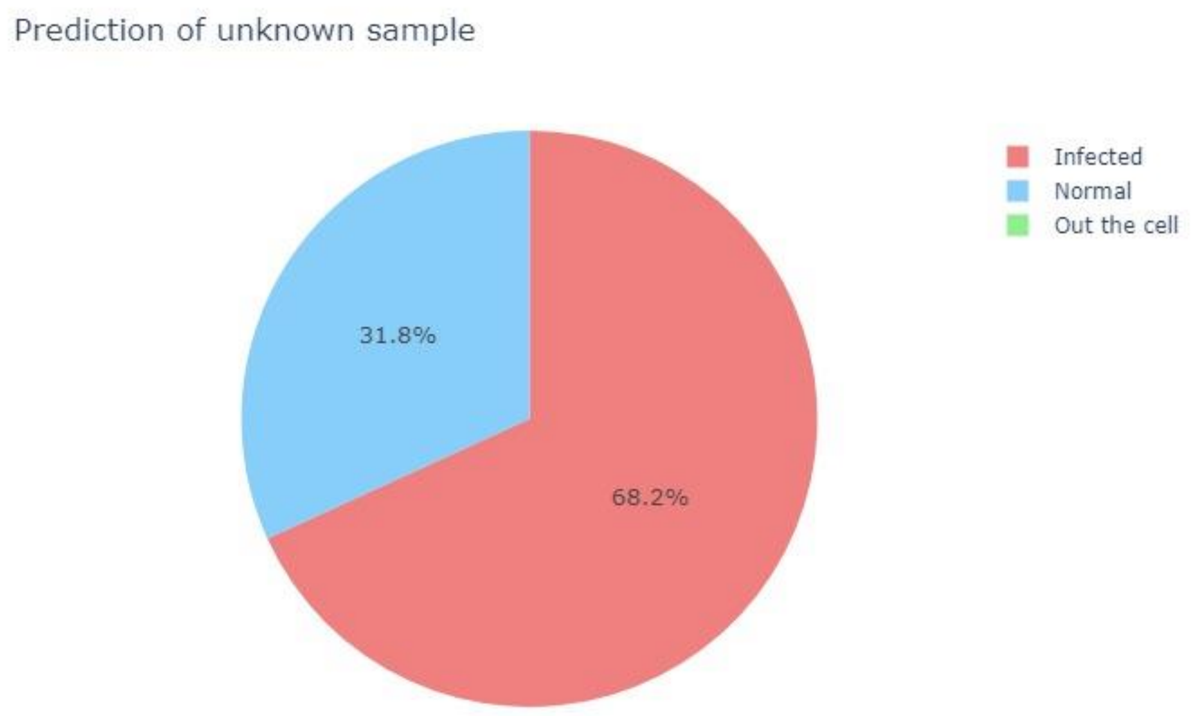

Blind test sample No.3

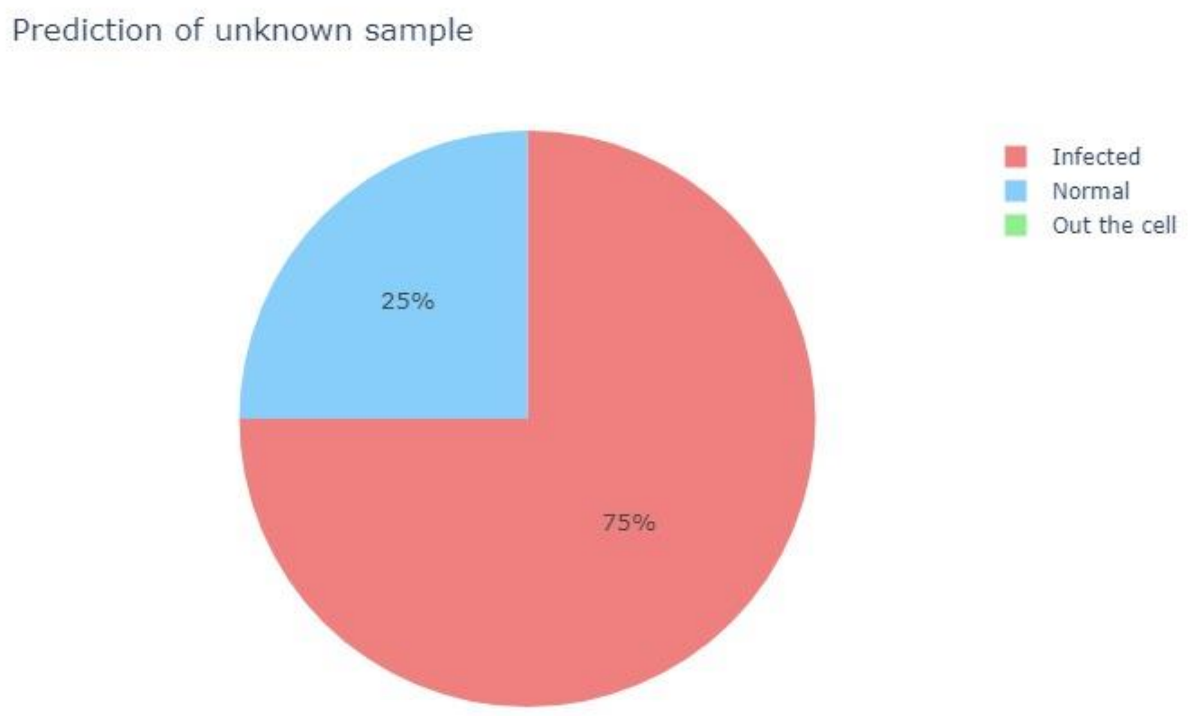

Blind test sample No.4

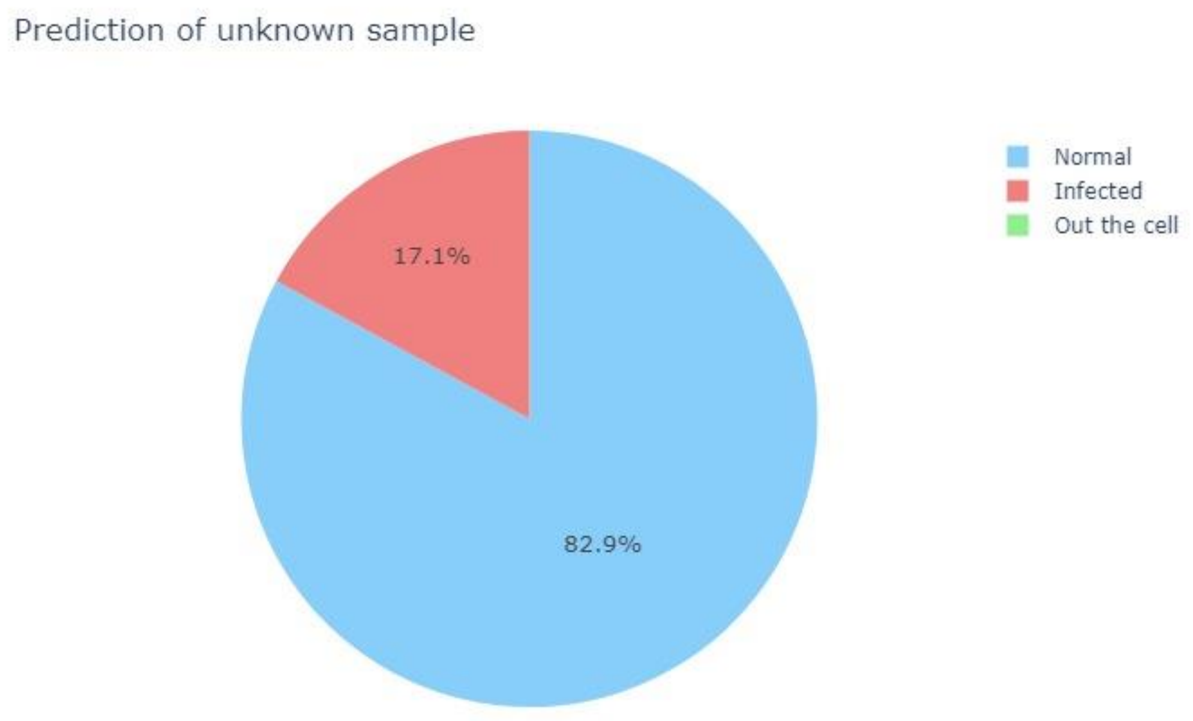

Blind test sample No.5

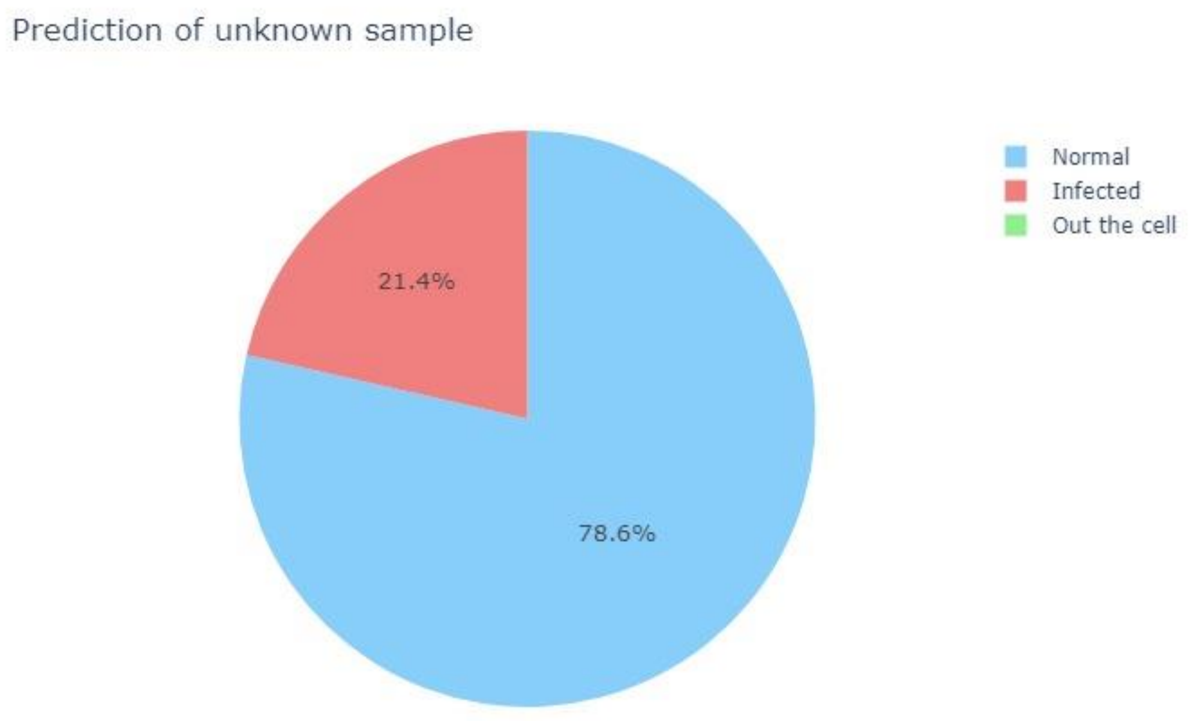

Blind test sample No.6

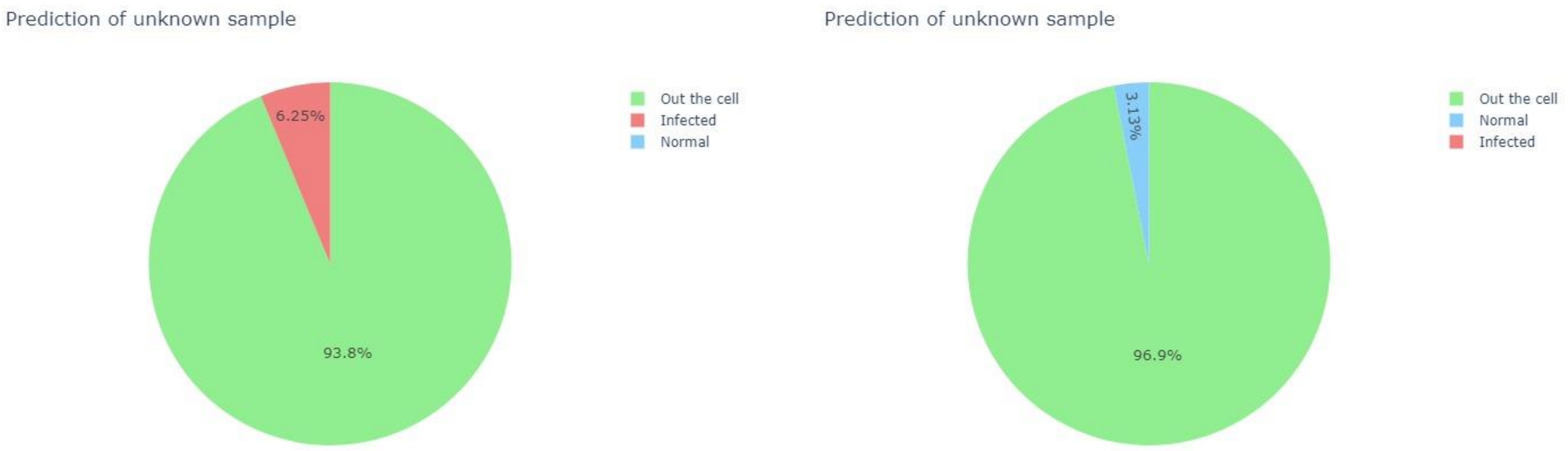

Blind test sample No.7

Blind test sample No.8

Figure S1. pie charts of the blind data sets, Supplement for Figure 10.
